# The Connections between Attitudes towards Nursing Home Placement, Intergenerational Solidarity, and the Conflict between Tradition and Modernity among Three Generations of Arab Muslim Families in Israel

**DOI:** 10.1155/2021/6148980

**Published:** 2021-08-23

**Authors:** Pnina Ron

**Affiliations:** School of Social Work, University of Haifa, Haifa, Israel

## Abstract

The goal of this study was to examine three generations of Arab Muslims in Israel, to investigate the relationships between their attitudes regarding the placement of an older relative in a nursing home, intergenerational solidarity, and to ultimately proceed with the nursing home placement. The backdrop to this examination was the increasing sociocultural tension between modernization tendencies and the long-established traditions and norms in the Arab Muslim society in Israel. The sample included a total of 126 university students, as well as one parent and one grandparent of each student. All participants completed identical questionnaires examining the attitudes towards the nursing home placement of an elder relative. The findings of the study indicate a strong objection among the youngest generation, whose attitudes were more similar to those of their grandparents than to those of their parents. Psychosocial mechanisms in the Arab Muslim population, such as intergenerational solidarity, has been the subject of increased scrutiny and debate over recent years, given the intensive pace of modern developments, which has called into question the familiar norms, thus constituting a threat to the tradition that has guided the population throughout numerous centuries and generations.

## 1. Introduction

Nursing home placement became an official part of the welfare services available to older individuals in the Arab sector in Israel approximately 30 years ago [1]. This phenomenon evolved at the same time that the Arab society in Israel was undergoing changes in several fields, including, society, economy, education, proprietorship, as well as the distribution of authority roles within the family [2]. According to the Arab tradition, aging people's main support network is the family, and typically, reliance on the assistance provided to the community by official services is either minimal or nonexistent. Arab society accords a great deal of significance to maintaining family solidarity and particularly honoring one's parents and elders [3, 4]. According to this tradition, the family is responsible for caring for the older relative, and consequently, the informal support network in the Arab sector is remarkably strong. Data regarding support patterns indicate that the degree of support granted to old people in the Arab population is much higher than that granted to old people in the Jewish population. Furthermore, findings have shown that adult Arab children who observe the tradition find it rewarding to care for their aging parents, whereas the youngest and less traditional generation attributes less importance to family solidarity [5–7]. Consequently, placing an older parent in a nursing home is perceived as shaming the family, and those who opt to avail themselves of such services are viewed as having betrayed their parents [8].

In recent years, however, there has been a shift in the way that the nursing home is perceived among the Arab population, a shift which is related to the changing attitude in Arab society towards the trends of modernization.

The first nursing home for the Arab sector was established in 1992 by the ESHEL organization in the village of Dabburieh. Currently, there are a substantial number of nursing homes for the Arab sector in Israel; most of them are located in northern Israel, where the largest concentration of Arab Moslems resides. In the past decade, there has been a significant increase in the percentage of older people in Israel. In the Jewish sector, older adults constitute 11% of the population, compared with 3% in the Arab sector. The Arab population, in general, is younger than the Jewish population in Israel, with the number of people older than 65 years totaling 70,350. Furthermore, among the Arab population of age 65 years or older, 35% are older than 75 years, compared with 47% in the elder Jewish population [9]. The rate of disabilities among the elder Arab population is relatively high, whereas the rate of nursing home institutionalization is low, as manifested in the relatively small number of official institutions available to this sector, such as nursing homes, activity centers, and senior citizen centers [2]. The percentage of older Arab adults living in nursing homes is 0.9%, compared with 6% in the Jewish sector, and of these, approximately half resides in northern Israel where they constitute 30% of all of the aging population, regardless of sector affiliation [9]. The paucity of professional literature regarding older adults in the Arab sector, their families, and their placement in nursing homes is evident; moreover, the focus of studies conducted in this field to date has been on Arab society as a whole, without examining attitudes within the Arab sector or within the Arab family unit.

### 1.1. Intergenerational Solidarity

The intergenerational solidarity model is based on the exchange theory [10, 11] and is used by numerous researchers to examine intergenerational solidarity, and especially intergenerational relationships of reciprocity, and the phenomenon of transmission between aging parents and their children [10, 12]. The model, which introduces a new point of view for the exchange theory, is intended to provide a comprehensive explanation of the relationships among generations in a single family, by assessing the quality and characteristics of these relationships. In this model, intergenerational solidarity is perceived as a multidimensional phenomenon, which includes six components that reflect the reciprocal relationships in a family [10, 11, 13].

1. Structural solidarity refers to residential patterns, the geographical distance between generations and the number of generations in the family
2. Associational solidarity is about the number and patterns of intergenerational interactions
3. Affectual (emotional) solidarity describes the quality of the intergenerational bond, expressions of love, affection, and emotional support
4. Consensual solidarity reflects the degree to which generations in the family agree on issues of social and political values
5. Functional solidarity concerns the degree of assistance and instrumental support between generations and the reciprocity of such support
6. Normative solidarity the degree of commitment to family roles and norms

The researchers [13] described the findings from the Israeli portion of a broad (Oasis) survey that examined intergenerational relationships and transmission in several countries (Israel, Norway, England, Germany, and Spain) using the intergenerational solidarity model [14]. They found that the majority of participants from all countries reported a high level of normative solidarity, although the solidarity level reported among Israeli participants was higher than that reported by participants in Spain or Germany. According to these researchers, the explanation is based on the fact that in Israel, there is a greater emphasis on family solidarity and a stronger commitment to supporting aging parents than in other countries and that this finding is particularly related to input from Arab families in Israel.

In the current study, only four (affectual/emotional, functional, normative, and structural solidarity) of the six dimensions of the model were used to examine and gain a better understanding of the essence of the intergenerational relationships and family bonds as perceived by aging individuals, their children, and their grandchildren in the framework of the Arab Muslim family and especially to gain insight into the attitudes of the three generations in the Arab family towards nursing home institutionalization of an aging family member.

### 1.2. The Phenomenon of Nursing Home Placement among the Older Adults in the Arab Society

Despite the generally negative attitudes among the Arab population regarding nursing home institutionalization, the approach of older people and their relatives towards the formal services available suggests a mixture of traditional and modern perspectives [15]. In a study that examined the attitudes of Arab families that decide to place an aging family member in a nursing home, findings indicated that participants found it easier to see an aging relative in an institute such as a hospital, as it demonstrates to their society that this is a temporary arrangement for the purpose of receiving emergency care, so that the aging relative can be brought back into the home [16]. A different study that sought to gain insight into the reasons for the high rate at which nursing home residents in Dabburieh were leaving the nursing home found that the cause was mostly the need to abide by social norms of caring for older family members, due to a sense of shame, and social pressure [8]. As a result of the attitude of the Arab society towards nursing homes, in many cases, families consider the option only after it is too late, as the older relative experiences health complications and reduced functionality to such an extent that the family can no longer cope with the increasing problems and needs [17]. Placing an aging relative in a nursing home is likened to consumption by necessity and the fact that numerous people who reside in nursing homes are so averse to it as to leave even after a relatively long stay suggests that this institution is not well accepted. Such a negative attitude is characteristic of Arab society, as is a lack of awareness of its advantages [17]. Researchers have found that the existence of a nursing home that is adapted to fit the social and cultural needs of the Arab Muslim population increases awareness and the family's willingness to be assisted by this system. Thus, once they become familiar with the system, the typically negative attitude of Arab family members tends to change: the system is seen as a positive option, families wish to let their aging relative reside in a nursing home for a prolonged period, while continuing to maintain an active role in the life of their relative [18].

Attitudes towards a particular behavior are a function of the personal component, i.e., the individual's positive or negative assessment of the relevant behavior in relation to a given set of circumstances; in this case, we are considering the behavior of placing an aging relative in a nursing home. The familial and cultural Muslim norms in Arab society regarding this particular behavior inevitably influence the individual's perception of the issue. The theoretical framework for the current study is based on the theory of reasoned action [19]. This theory constitutes the framing model for assessing the relationship between beliefs, their underlying assumptions and the individual's attitude, and whether this relationship can serve to predict social behavior.

The focus of the current study when examining family members' attitudes regarding nursing home institutionalization of an aging relative was on three generations in a family: the aging person, the middle generation, and the grandchildren's generation. This examination took into account the extensive changes that the Arab society is undergoing in terms of its familial and social structures.

Based on the information reviewed herein, two major study hypotheses emerged:

1. The attitudes of the three generations of family members regarding nursing home institutionalization of an aging parent or grandparent will vary by age, such that the younger the participants are, the stronger will be their support in favor of this move.
2. A negative correlation will be found between the quality (relative strength or weakness) of intergenerational solidarity and the attitudes of the three generations of family members regarding the placement of an aging parent or grandparent in a nursing home; specifically, the younger family members will exhibit a weaker solidarity and a more positive attitude towards the placement of an aging parent or grandparent in a nursing home, whereas members of the older generations will exhibit stronger solidarity and less positive attitudes towards such placement of an aging family member.

## 2. Methods

The study was approved by the Ethics Committee of Haifa University.

### 2.1. Population and Sampling

The study population consisted of three generations of Muslim Arabs living in the northern region of Israel: an elderly family member (grandparent generation), his or her adult child (parent generation), and members of the youngest generation, specifically 126 undergraduate students attending Haifa University (grandchild generation). The population was recruited using the convenience sampling method.

### 2.2. Data Collection

Each student was given three identical questionnaires, one to be completed by the student and the other two for a parent and a grandparent of the same gender as theirs. After the goals of the research were explained, all of the participants in the sample signed an informed consent form, indicating their willingness to participate in the research voluntarily.

Table 1 includes demographic data regarding participants' characteristics. The students demonstrated enthusiasm at the opportunity to examine the issue of attitudes within their families. Nonetheless, it was not always possible for them to recruit family members of the same gender as theirs. As a result, it was decided that rather than conducting intrafamilial statistical analyses, a generational cross-sectional analysis of the data would be conducted. A total of 140 triads of questionnaires were distributed; of these, 14 triads were rejected, for the most part, due to incomplete questionnaires pertaining to one of the generations in a family.

An examination of the data presented in Table 1 indicates that the majority of participants (63%) were women, the grandparent generation had the largest number of children (5.84, SD = 2.27) and that the lowest level of education among all three generations was 6.02 years of study (SD = 5.05).

### 2.3. Instruments

The instrument used in this study was a structured questionnaire, which had been translated from Hebrew into spoken Arabic by a professional translator and then back translated into Hebrew by two bilingual researchers. The questionnaire consisted of four parts, as follows.

1. A Demographic Questionnaire collected information about background variables of each participant. This section was designed specifically for the purposes of the current study.
2. An Attitudes Questionnaire was intended to examine participants' general attitudes regarding nursing home placement. This variable was measured according to the instrument designed by Tzivoni [20], which addresses two dimensions: (a) attitudes that favor nursing home placement and (b) attitudes against nursing home placement. This section of the questionnaire was selected based on studies that indicate the existence of a positive relationship between attitudes and actual actions taken and the ability to predict actions based on attitudes. Participants indicated their responses to statements using a Likert-like scale, ranging from 1 = completely agree to 7 = completely disagree. Of the 38 statements in this section, approximately half represent attitudes in favor of nursing home placement and half represent attitudes against it. Reliability measures were as follows: for attitudes in favor of nursing home placement, Cronbach's alpha = .63; for attitudes against nursing home placement, Cronbach's alpha = 61.
3. The Intergenerational Solidarity Questionnaire is based on Bengtson and Schrader's [21] instrument for measuring intergenerational solidarity and conflict. The original instrument includes 72 statements intended for parents and children. In this current study, solidarity was measured in terms of the following four dimensions: (a) affectual (emotional), (b) functional, (c) normative, and (d) structural solidarity and included 58 statements. The instrument was translated into Hebrew and adapted to the needs of the Oasis study, which was conducted by a collaborative team of senior researchers from five different countries [13]. For the purposes of the current study, the questionnaire was shortened to 54 items. Participants indicated their degree of agreement with each item using a seven-point Likert-like scale, ranging from 1 = completely agree to 7 = completely disagree.
4. The reliability measures found for each dimension of the Solidarity Questionnaire ranged from .61 to .91, Cronbach's alpha. In the current study, the average score for all solidarity types is presented as a single variable, referred to as “the quality of intergenerational solidarity,” whereby a low score indicates weak solidarity and a high score indicates strong solidarity.
5. Predicting Social Behavior (PSB) Questionnaire was based on the instrument designed by and Ajzen and Fishbein [22] and was adapted for the specific purposes of the current study, namely, predicting the intent to place a relative in a nursing home. Participants indicated their agreement to 27 statements using a Likert-like scale, ranging from 1 = completely agree to 5 = completely disagree. The statements in this questionnaire were related to three dimensions: (a) what nursing home placement means to the respondent (nine statements); (b) willingness to place a relative in a nursing home in the case of the relative's loss of functional autonomy (nine statements); and (c) willingness to place a relative in a nursing home in order to ease the family's burden of care (nine statements). In the course of the statistical analysis, it was found that the three dimensions were interrelated and demonstrated a high reliability score, both for the entire sample and for each of the generations separately. Consequently, it was decided to consider the entire instrument as a single variable, instead of measuring each dimension separately. The reliability of the entire instrument was Cronbach's alpha = .69. From here on, this behavior that this questionnaire was meant to predict will be referred to as “the intent to place a relative in a nursing home.”

## 3. Results

The current study examined the relationship between personal variables (attitudes regarding nursing home placement and the intent to place an aging relative in a nursing home), interpersonal variables (intergenerational solidarity), and demographic variables (e.g., age, education, religiosity).

In the first stage of the analysis, distributions, means and standard deviations were calculated for all of the variables related to nursing home placement per each generation (see Table 1). A review of the data in Table 1 indicates that the highest scores were assigned by the grandparent generation, indicating a positive attitude towards nursing home placement (*M* = 3.89, SD = .48) and the intent to move into a nursing home (*M* = 3.37; SD = 0.54). In other words, the positive attitude regarding nursing home placement was strongest among the grandparent generation, and this generation also indicated the strongest intent to place a relative in a nursing home in the context of “loss of functional autonomy”.

To examine the internal connections among the nursing home placement variables, a total score was calculated based on the mean score for each of the research variables. In addition, Spearman's tests and Pearson's tests were conducted to examine these connections. These analyses were conducted using the entire sample. There is room to examine whether there is a connection when generational division is not taken into account.

The data presented in Table 2 indicate that no significant positive correlation was found between the variable of education and any of the other relevant study variables. Findings of the study indicate no significant relationship between participants' education level and either their attitudes towards nursing home placement or their intent to place a relative in a nursing home.

The variable of religiosity correlated negatively with the attitude favoring nursing home placement (*r* = −.34, *p* < .05) and with the intent to place an aging relative in a nursing home in case of loss of functional autonomy (*r* = −.22, *p* < .05). Furthermore, a strong positive correlation was found between the variable “arguments favoring nursing home placement” and the variable of intent to place a relative in a nursing home due to loss of functional autonomy (*r* = .63, *p* < .01).

To examine the relationship between the variable of family status and the attitudes regarding nursing home placement among the three generations, Chi square values were calculated. No significant correlation was found between family status and attitudes for or against nursing home placement. In contrast, strong and significant correlations were found between the level of religiosity and attitudes against nursing home placement, both among the generation of grandchildren (*χ*^2^_(34)_ = 53.17; *p* < .01, rc = 0.8; *p* < .01) and the generation of grandparents (*χ*^2^_(33)_ = 50.03; *p* < .01, rc = 0.7; *p* < 0.01).

At this stage, and to examine the research hypotheses, several two-way ANOVA procedures were conducted. For the purpose of statistical analysis, relevant variables were grouped into one of two categories, either above or below the mean score value for each variable. Findings of these calculations indicated the following.

- The generation variable had a significant effect on the mean score for attitudes against nursing home placement (*F*_(2,162)_ = 5.36, *p* < .01). To examine the source of this effect, a Scheffe test was conducted, which revealed that the mean score for attitude opposing nursing home placement was higher among the grandchild generation than it was among the grandparent generation. On the attitude variable of opposition to nursing home placement, no significant difference was found on the intent variable between the grandparent and the parent generations or between the parent and the grandchild generations.
- The generation variable had a significant effect on the mean score for intent to place an aging relative in a nursing home (*F*_(1,162)_ = 12.9, *p* < .01). To examine the source of this effect, a Scheffe test was conducted, which revealed that the mean score for intent to place an aging relative in a nursing home was higher among the parent generation than among the grandchild generation. No significant difference was found on this variable between the grandparents and the parent generations or between the grandparent and the grandchild generation.
- The generation variable was found to have a significant effect on the intergenerational solidarity variable (*F*_(2,162)_ = 11.58, *p* < .01). A Scheffe test revealed that the mean score for intergenerational solidarity was higher among the grandparent generation than among the parent generation. No significant difference was found between the mean scores on intergenerational solidarity between the grandparent generation and the grandchild generation or between the parent and grandchild generation.

Up to this point, findings of the current study indicated that some of the variables were related to positive attitudes regarding nursing home placement, as well as to opposition to placing a relative in a nursing home. To summarize the findings and examine the first research hypothesis, a multiple regression analysis was conducted. Findings of this analysis are presented in Table 3.

An examination of the data in Table 3 reveals that in the case of a positive attitude towards nursing home placement among the grandparent generation, the remaining variables explained 32% of the variance, but only the variable of religiosity significantly predicted positive attitudes towards nursing home placement, and this regression model was found to be significant (*F*_(7,45)_ = 3, *p* < .05). In the case of a negative attitude regarding nursing home placement, it was found that among the grandparent generation, the variables of the model explained 8% of the variance; however, the regression model did not render a significant outcome (*F*_(7,45)_ = 0.58, n. s.).

Focusing on the parent generation, it was found that when the dependent variable was positive attitudes towards nursing home placement, 16% of the variance was explained by the other study variables, but the regression model did not render a significant outcome (*F*_(7,45)_ = 1.25, n. s.). However, when the dependent variable was a negative attitude towards nursing home placement, 29% of the variance was explained by a single variable, namely, intergenerational solidarity, and the model rendered a significant outcome (*F*_(7,45)_ = 2.64, *p* < .05).

An examination of the grandchild generation found that when the dependent variable was positive attitudes towards nursing home placement, the variable of religiosity predicted 48% of the variance, and the regression model was significant (*F*_(7,45)_ = 4.37, *p* < .01). The regression model was significant also when the dependent variable was negative attitudes regarding nursing home placement (*F*_(7,45)_ = 5.32, *p* < 0.01). Both the variable of intergenerational solidarity (*β* = 0.38, *p* < .05) and the variable of intent to place a relative in a nursing home (*β* = −.60, *p* < .01) significantly predicted attitudes against nursing home placement, with the latter variable being the more significant among the two.

To summarize, the findings of the regression analysis indicated that the variable of religiosity significantly predicted a positive attitude regarding nursing home placement among the grandparent and the grandchild generations; none of the variables predicted a positive attitude among the parent generation. In other words, the higher the level of religiosity was, the less positive were the attitudes regarding nursing home placement, such that higher orthodoxy was negatively associated with favorable attitude regarding nursing home placement.

By contrast, as regards negative attitudes regarding nursing home placement in each of the generations, it was found that variable of intergenerational solidarity explained the negative attitude among both the grandchild and the parent generations. As regards the grandchild generation, the variable of intergenerational solidarity had a negative effect on the negative attitude regarding nursing home placement, whereas the variable of intent to place a relative in a nursing home had a positive effect on the negative attitude.

## 4. Discussion

The goal of the current study was to examine and compare the attitudes of three generations (grandparents, parents, and grandchildren) in the Arab Muslim family in Israel regarding the placement of an aging parent or grandparent in a nursing home. According to the Arab tradition, the family that constitutes the major support network for older adults refrains from accepting the assistance of formal services offered by the state, such as nursing home placement for an aging relative. However, as the processes of change and modernization begin to affect the Arab society, they leave their mark on social values as well. The current study and its findings give rise to two major issues that merit discussion: the first is related to the nursing home placement of aging relatives in Muslim society and the second is the issue of collectivism versus individualism in the Arab Muslim society. It is important to emphasize that this society is undergoing two processes simultaneously: change and modernization on the one hand, and a return to tradition, on the other hand.

A detailed study that reviews nursing home placement of Muslim older adults in the United States claimed the following: “With Islam placing a strong emphasis on caring for one's parents in the home, along with cultural barriers, Medicaid and Medicare's focus on nursing homes is not a viable option for Muslim Americans” ([23]; p. 1). The author offered several possible causes that might explain why Muslim older adults living in the United States do not opt for nursing home placement, chief among them, the issue of religion. The Koran, which is revered as the word of God, states the following.

Your lord has decreed that you worship none but Him, and that you be kind to parents. Whether one or both of them attain old age in your life, say not to them a word of contempt, nor repel them, but address them in terms of honor.

Thus, the more religiously conservative the family is, the more likely it is to interpret the command to honor one's parents to mean providing unmediated care in one's own home for aging parents and relatives [24]. The researcher indicated that placing an aging Muslim parent in a nursing home is considered a type of elderly abuse. In her study, Al-Heeti [23] noted the discretion required in Muslim families when a relative requires medical attention or treatment, so that the family can avoid public shame or humiliation, which stem from the social norm that views the family—rather than the public services—as responsible for caring for an aging relative. As the author explains, it is important that the family appear to be the one taking care of the aging relative, and it is crucial to maintain discretion whenever a member of the Arab Muslim family in the United States requires treatment outside the home. A further complication stems from the fact that an aging Muslim man might reject treatment from a female healthcare professional, while an aging Muslim woman might be apprehensive or feel uncomfortable being left alone to be cared by a male healthcare professional. These issues are familiar in Israel as well, yet they have never been directly studied. This is because, given Israel's small geographic area and the particularly the geographic concentration of Arab Muslim populations in Israel, Muslims of any generation would be reluctant to divulge collaborate with researchers by divulging information on such sensitive issues.

The transition from a traditional society to a modern one is referred to in the professional literature as social mobilization [7]; a concept that conveys a transition from a generations-old patterns of belief and behavior to newer ones. In recent years, their tradition has been subjected to abrupt changes. The modern Western philosophy has led traditional societies to be characterized as different, “exotic and also as reflecting lower stages of evolutionary advancement”. As a result of this social perception, traditional societies in the United States, India, Iran, and other countries have opted to adjust and adapt to the Western philosophy, which conveniently dictates behavior patterns but does not interfere with beliefs or ideologies. A lot of it is related to the quality of life offered to the elderly and how much their children are satisfied from it. Nonetheless, the encounter between these two worlds creates an ideological crisis, and the need to cope with the conflict between modernity and tradition is not without problems [25]. The Arab population that now resides in the state of Israel was under the Turkish rule when modernization began, and later under British rule, until 1948 when the state of Israel was established. Israel has followed the Western philosophy and its behavior patterns and, as the world of computers and digitalization and the notion of the *global village* are increasingly becoming the norm, Arab families, and especially Muslim Arabs, have found themselves faced with the choice of either adapting to the new world or withdraw and isolate themselves from the surrounding society. Consequently, psychosocial mechanisms, such as intergenerational solidarity and transmission, have been used in recent decades as a way of coping with the new and evolving reality, which is increasingly affecting aspects of life that for generation upon generation were the accepted and familiar norms [8, 26].

### 4.1. Discussion regarding Hypothesis 1

The current study, examined the attitudes of 126 university students, one of their parents and one of their grandparents, thus reviewing three generations of Arab Muslim families. The goal of the study was to gain insight into the participants' attitudes regarding nursing home placement and to compare these attitudes among the three different generations. Two hypotheses guided this study: the first was that the three generations would differ in their attitudes regarding nursing home placement of an aging parent, such that the younger the participants are, the stronger will be their support in favor of this move. This hypothesis was not confirmed; on the contrary, findings indicated that the younger the participants' age, the greater the opposition to placing a parent or grandparent in a nursing home. This was perhaps the most surprising of the findings of the study, as the grandchild generation's attitudes against nursing home placement were stronger than those of the other two generations. Furthermore, they reported a low level of intent to place an aging relative in a nursing home, particularly if the older relative has lost some degree of functional autonomy. Although the education level of the youngest generation was also the highest, and the expectation was that the education variable would have the strongest effect on favoring nursing home placement, this expectation was not confirmed. Moreover, reports from the youngest generation indicated a higher level of religiosity. It is possible that this indicates a desire to hold on to a tradition that is fading. The attitudes of the youngest generation were more similar to those of the grandparent generation than to their parents' generation, which suggests that the intergenerational transmission did not play a role in this sample.

A study that examined filial responsibility among three generations of Arab Muslim families [27] found positive attitudes and perceptions among all three generations towards the notion of filial responsibility and assisting aging parents, with the youngest generation's positive attitudes ranking higher than those of their parents or grandparents. Findings also demonstrated that in all three generations, women's attitudes were more positive than those of men, and the attitudes of religious participants were more positive than those of their secular counterparts. A study that examined the experiences of Muslim families who have a family member in a nursing home found that psychological attitudes and sociocultural norms are two challenging issues affecting families both in the decision making about nursing homes stage and living with this decision after making it [28].

A possible explanation for the attitude among the youngest generation, as found in the current as well as in the aforementioned study, may stem from the tendency to associate increased religious practice with the strengthening of traditional social norms. An increasing inclination towards conservative religiosity has been noted in the Arab world in general and among Muslims in particular. Not surprisingly, this trend has emerged also among Muslims in Israel and is noticed among the youngest generation [23, 28, 29].

In the current study, the assumption was that intergenerational transmission would be the psychosocial mechanism by which traditional cultural and religious norms were inculcated to younger members of the family unit. Intergenerational transmission is a concept that indicates the finding of similarities between consecutive generations in terms of attitudes and behavioral patterns, which seemingly are passed down from generation to generation. Some researchers [30, 31] have used the term *behavioral genetics*. People are generally affected directly by the familial model within which they were raised, whereas the models provided by previous generations have an indirect and subconscious effect. Consequently, traditions, behaviors, beliefs, and rules are passed from generation to generation [32, 33]. The researchers claimed that the family unit exerts horizontal and vertical pressures on its members; the vertical pressures, which are passed from one generation to the next, include general approaches and ways of functioning and solving emotional problems. Thus, vertical pressures may be related to adult children's sense of responsibility and commitment to care for their aging parents, and they also account for the parents' expectation that in due time and according to the tradition, they too will be cared for by their children. Thus, these vertical pressures provide a possible explanation not only for the current study's finding of pervasively negative attitudes regarding nursing home placement among all participants but also for the exceptionally strong opposition among the youngest generation. Rijnaard et al. [34] suggested that family closeness and involvement before the initialization will have a major impact on the attitudes, willingness, and involvement in the process of nursing home placement of an elderly family's member.

The current study's findings indirectly indicate the breaking of this psychosocial pattern. None of the findings of this study—whether related to the effects of demographic variables (education, religiosity), to participants' attitudes, to the intent to place a relative in a nursing home in the case of loss of autonomy, or to the variable of intergenerational solidarity—indicated any form of consecutive intergenerational transmission. As regards the education variable, there is a simple explanation for the absence of intergenerational transmission, namely, the fact that in contemporary advanced societies, members of the youngest generation are relatively more educated than either their grandparents or their parents, and this is also true in traditional Arab societies and is particularly noticeable among the women in the Arab society.

The finding that the highest level of religiosity was reported by the youngest generation contradicts the known patterns of intergenerational transmission. As previously mentioned, a possible reason for this may be the global phenomenon of increased religiosity among the youth of all three monotheistic religions. Furthermore, observing and adopting the role of caregiver to one's aging relatives is considered the result of learned behavior [35]; hence, the clan-like lifestyle shared by all of the generations in the Arab family could serve to explain the similarity of attitudes and intent regarding nursing home placement between the grandparents' and the grandchildren's generations.

Notwithstanding, members of the youngest generation in Arab Muslim families in Israel move out of the family home to pursue advanced studies, and occasionally, they even move overseas. Due to the rate of unemployment in Israel, some of them leave their homes located in rural areas and move to major cities, where the chances for finding employment are much better. Despite the observation made by the author [36], it may be surmised that a high level of opposition among the youngest generation to the placing of an aging family member in a nursing home indicates the grandchildren's inability to understand the functional and health-related needs of their grandparents. Members of the grandchildren generation view their grandparents in terms of the significant role they have in the extended family, rather than as older adults who need round-the-clock attention to a greater or lesser degree. In other words, their attitudes are based on emotions rather than on rational considerations.

### 4.2. Discussion regarding Hypothesis 2

The second hypothesis was that a negative correlation would be found between the strength of intergenerational solidarity and the positive attitudes of members of the three generations regarding nursing home placement. This hypothesis was based on the assumption that the youngest generation would be more open to the idea of nursing home placement than the oldest generation, and hence, the youngest generation would represent the greatest change in terms of accepting modern behavior patterns. Such greater openness could be assumed, given that modern Arab society in Israel clearly understands that demonstrating concern for an aging family member but confining care and support to the family framework do not go hand in hand. Not only is such an approach liable to be detrimental to the health maintenance of the aging relative, it would also hinder the ability of the young Arab family to advance and develop in today's modern competitive and capitalist world [37, 38]. However, the second hypothesis was not confirmed, and as in the case of the first hypothesis, the hypothesis was contradicted by the findings.

In the current study, it was found that the generation of grandparents assigned higher scores and thus indicated a stronger agreement with the notion of nursing home placement compared with the grandchildren's generation. They also indicated a higher degree of intent to move into a nursing home if they suffer a loss of autonomy. This unexpected gap may express the grandparents' realistic understanding that the organized healthcare system is better equipped to provide significant medical assistance, monitoring their dietary and activity needs on a regular basis as is the practice in nursing homes. Another possible explanation for this unexpected finding, which was found to be related to the level of intergenerational solidarity, maybe the grandparents' effort to avoid becoming a burden on the younger family members, particularly on the women, who in recent years have ventured beyond the home and found suitable employment [35].

As regards the middle generation, it appears that the more the members of this generation reported having an affectionate relationship with their parents, meeting with them, calling them, and providing emotional support, the more likely they were to display attitudes and claims in favor of nursing home placement. By contrast, when the members of the middle generation reported providing essential functional care for their parents, in terms of financial assistance, shopping, and housekeeping, all of which necessitated living in close proximity to their parents, the less likely they were to display attitudes favoring nursing home placement. The findings of the study demonstrate the devotion of the children and their commitment to care for their parents. Indeed, this is the norm in the Arab family, where all the generations live in close geographic proximity and the large number of children and grandchildren ensures that there is always someone available to care for the aging family members, visit them, address their needs, or live with them in the same household. In addition, this high degree of devotion may reflect the religious and cultural values that characterize the desired filial behavior of children towards their aging parents. Thus, it is possible that these findings indicate, to some degree, that the devotion of the middle generation towards their parents is limited, in the sense that they understand that should intensive long-term treatment become necessary, reliance on the available formal resources for assistance would not necessarily be considered a betrayal of their filial commitment.

As regards the grandchildren's generation, much like the perspective of the grandparent generation, the quality of their relationship with their grandparents was not related to their degree of agreement with an attitude favoring nursing home placement. Rather, their attitude against nursing home placement was related to the degree to which they reported receiving financial and emotional assistance from their grandparents and the degree to which they felt obligated to give back something in return for said assistance. Grandchildren also reported that the better their relationship with the grandparent was, the less inclined they were to place him or her in a nursing home in the case of loss of autonomy. The grandchildren attributed less significance to the advantages associated with nursing home placement (such as lightening the family burden, availability of medical services, relative independence for the aging grandparent, and more comprehensive support available) and greater significance to the cultural norm, which views nursing home placement as unacceptable. It appears that the intergenerational loyalty and commitment that even the grandchildren expressed towards the oldest generation led them to believe that they could fulfill all of the necessary functions and thus avoid nursing home placement. The strong emotional bond was created by the fact of living in a joint residence, and thus spending a great deal of time with the grandparents is a likely explanation for the grandchildren's sense of commitment towards the grandparents. In the current sample, a large percentage of the grandchildren indicated that they were living or had lived with a grandparent, in most cases with a widowed grandmother who feared living alone or who could not be accommodated in her sons' homes. It may be surmised that the grandchildren's sense of responsibility and commitment towards their grandparents consisted mostly of emotional and less of functional support, which alone is not sufficient to ensure that the aging grandparent receives appropriate care in the long term.

### 4.3. Limitations of the Study

The main limitation of the current study is related to sample and sampling. Recruiting members from three different generations of Arab Muslim families constitutes a rather complex logistical challenge. The willingness of university students to engage in this study and to learn about their families was a facilitating factor, but it constituted a methodological limitation.

Future studies should continue this line of investigation by conducting cross-sectional research of the Arab Muslim population in Israel, so as to characterize populations in various geographical regions, differentiating between the rural villages and major Arab cities, as well as between the population in the major cities and the Bedouin communities living in unrecognized villages (known as P'zura, meaning, scattered dwellings). This type of differentiation will make it possible to determine the degree to which each group is exposed to the various healthcare options available to aging family members.

## 5. Summary

A review of the current study should take into account global trends related to longevity and quality of life. The age at which individuals choose to move to a nursing home is constantly rising, both in Israel and throughout the world. Improved quality of life during old age postpones the decision and delays the actual move, which in turn means that in the Western world, a smaller percentage of the aging population opts to live in nursing homes. In Israel, this delay is related also to the tight family structure that characterizes Israeli society in general. Hence, compared with other Western countries, there was never a sudden upsurge in the number of people moving into nursing homes, and even to this day, the trend is rising only gradually, and the decision is typically postponed for later years.

In addition to the global and local trends, also the particular characteristics of older adults in the Arab society in Israel should be examined, as these tend to differ compared with the Jewish population. Thus, for example, in the Arab society, the average age of the older adult population is typically younger than that in the Jewish society, and a higher percentage of the aging population in the Arab society requires medical care (30%) compared with the older adult population in the Jewish society (14%), often because of disabilities that require daily attention.

Despite the changes that the Arab society in Israel is undergoing, there is still an emphasis on the importance of taking responsibility for caring for aging family members and on treating them—as much as possible—with the respect they are due.

## Figures and Tables

**Table 1:** Background characteristics (*N* = 378).

Generation	Variable	*N*	%
Grandparents	Number of participants	126	33.3
Parents		126	33.3
Grandchildren		126	33.3
Entire sample	*Gender*		
	Male	102	37.0
	Female	276	63.0
Entire sample	*Family status*		
	Married/living with a spouse	299	79.1
	Unmarried/live alone	77	20.9
Entire sample	*Level of religiosity*		
	Orthodox	102	27.0
	Conservative	165	43.6
	Secular	111	29.4
	Age	*M*	SD
Grandparents		76.48	6.71
Parents		49.86	5.78
Grandchildren		23.29	3.45
Grandparents	Years of education	6.02	5.05
Parents		11.18	4.4
Grandchildren		14.55	2.39
Grandparents	Number of children	5.84	2.27
Parents		3.47	1.39
Grandchildren		0.89	0.5
Grandparents	Attitudes towards nursing homes	3.89	.48
Parents		3.52	.54
Grandchildren		3.05	.60
Grandparents	Attitudes against nursing homes	2.75	.51
Parents		3.37	.88
Grandchildren		4.10	1.13
Grandparents	Intergenerational solidarity	5.42	1.15
Parents		3.69	1.39
Grandchildren		3.51	1.61
Grandparents	Predicting the intent to place a relative in a nursing home	3.37	1.95
Parents		4.93	1.26
Grandchildren		2.58	1.87

**Table 2:** The connections between the research variables^*∗*^.

	1	2	3	4	5
(1) Education	—	—	—	—	—
(2) Level of religiosity	−.050	—	—	—	—
(3) Attitudes towards nursing homes	.016	−.34^*∗*^	—	—	—
(4) Attitudes against nursing homes	−.024	.69^*∗∗*^	−0.04	—	—
(5) Intergenerational solidarity	−.050	.47	−0.15	0.27	—
(6) Predicting the intent to place a relative in a nursing home	−.033	−.22^*∗*^	0.63^*∗∗*^	−0.48^*∗*^	−.30

For the religiosity variable, a Spearman's correlation was conducted. For the other variables, a Pearson's correlation was conducted. ^*∗*^*p* < .05; ^*∗∗*^*p* < .01.

**Table 3:** Multiple regression analysis measuring the contribution of the research variables attitude for or against nursing home placement.

Generation/attitude	Grandparents		Parents		Grandchildren	
Variable	For	Against	For	Against	For	Against
Gender	0.04	−0.2	0.17	−0.06	0.2	−0.4
Family status	−0.23	0.05	−0.15	0.11	−0.3	0.26
Education	−0.01	−0.28	0.34	−0.19	−0.54	0.3
Religiosity	−0.09^*∗*^	0.24	−0.16	0.13	−0.68^*∗∗*^	0.45
Intergenerational solidarity	0.13	0.25	−0.27	0.28^*∗*^	−0.19	0.38^*∗*^
Predicting the intent to place a relative in a nursing home	0.18	−0.27^∗^	0.19	−0.21	−0.53	−0.60^*∗∗*^
Multiple*****R*^2^	0.32	0.08	0.16	0.29	0.48	0.58
*F*	*F*_(7,45)_ = 3^*∗*^	*F*_(7,45)_ = 0.58	*F*_(7,45)_ = 1.25	*F*_(7,45)_ = 2.64^*∗*^	*F*_(7,45)_ = 4.37^*∗∗*^	*F*_(7,45)_ _=_ 2.89^*∗∗*^

^*∗*^*p* < 0.05; ^*∗∗*^*p* < 0.01.

## Data Availability

The data will be available within a year/a year and a half upon request to corresponding author.
